# The Institutional Worker

**Published:** 1920-01-24

**Authors:** 


### INSTITUTIONAL WORKER

Being a Special Supplement to "The Hospital"

### OUR BUREAU OF INFORMATION.

### Rules for Correspondents.

1. Every letter must be accompanied by the coupon to be cut from the back cover (inside page) of The Hospital, current issue, and must contain the name and address of the correspondent with pseudonym for publication if desired. Replies by post cannot be given save under exceptional circumstances at the Editor's discretion.
2. Letters from Approved Homes in reply to special needs published in the Bureau should state terms and full particulars, and be sent prepaid under cover to the Editor of the Bureau with name written across coupon for identification.
3. Proprietors of Homes which have not yet been entered on the List of Approved Homes, but have spare accommodation likely to suit special needs, are invited to write for an application form for registration. The fee for registration, which includes two announcements of the Home in the Bureau and other privileges, is 10s.
4. All communications to be addressed to the Editor of The Hospital, 28 Southampton Street, Strand. London, W.C. 2, and marked "Bureau of Information."

### INSTITUTION FACTS AND FIGURES.

### POVERTY AND MATERNITY.

#### A SCHEME FOR HOME HELPS AND BOARDING OUT CHILDREN.

Dr. J. Wright Mason, the medical officer of health for Hull, has prepared an interesting scheme for extending maternity and child-welfare work in the direction of making arrangements for boarding out children during the mother's confinement in cases where home conditions are unsuitable, and for the provision of home helps.

He points out that in quite a large number of cases the home conditions of a lying-in woman are such as to be unsuitable for confinement, either by reason of the number of children, or of the need for some person to look after the house during this period. The Ministry of Health's suggestion that in some cases arrangements may be made for the children to be boarded out, infers the formation of a roll of suitable individual homes, and accordingly he suggests that the Health Committee should advertise with a view to securing such homes.

With regard to home helps, he mentions that the Education Committee has already arranged that some "helps" should be trained at the home-making centres, and proceeds to say that a home help should be preferably over thirty and under fifty-five. While suitability should be the primary object, it is desirable, where possible, to give the preference to widows and others who are entirely or mainly dependent on their own earnings. He says the following rules should be regarded as essential :—(1) Home helps must not refuse to go to the houses where they are sent. (2) Home helps must be clean, tidy, and punctual. They must wear an apron or overall. (3) They are forbidden to borrow anything whatever from the houses where they are working. (4) They are forbidden to receive any money from the person in whose house they are working. (5) In all matters of health, the home help must act under the orders of the nurse or midwife.

Regarding the provision of homes for children of widowed and deserted mothers and for illegitimate children, he mentions that a home for children has been established at Preston. The mother is expected to pay a reasonable sum (5*s.* per week if the father is unknown and the whole burden falls upon the mother, otherwise 4s. from the mother and 4s. from the father) towards the cost of supporting the child, which is maintained until about three years of age. The mother is encouraged to visit her baby and take an active interest in its welfare in the hope that at some time in the future she herself will be in a position to provide a suitable home. If the mother is unable to make some such provision, the child is admitted to an established institution.

Birmingham Corporation has not established a home for illegitimate children, but has contributed substantially to the cost of maintenance of homes of the type mentioned : one for illegitimate children and the other—a nursery training-school—which takes in children of mothers who have to enter hospital to undergo operations, or sanatoria, or who may have puerperal fever or other serious illness.

Dr. Mason suggests that the Hull Health Committee should invite applications from persons willing to act as foster mothers. He also suggests that an endeavour be made to obtain the names and addresses of persons residing outside the city who would be willing to offer board-residence for women after certain cases of confinement, and for children whose complete recovery after measles and whooping cough is retarded by the absence of a complete change of environment. Failing the offer of suitable places, there appears to be no alternative but for the authority to make its own arrangements and to provide for a small number of beds.

He further suggests that selected cases of married women (particularly those with large families), recommended by the clinical medical officer for a free midwife, be admitted to the Corporation's maternity home for their confinement, mentioning in this connection that the time is not far distant when the Committee will have to face the problem of providing for the confinement of unmarried women and girls. With the exception of the workhouses, the only place in Hull to which these persons may go is the maternity home in Linnæus Street. With the extension of the Corporation Maternity Home, it would be advisable to allot a portion for the reception of such cases, it being understood that abnormal cases would receive priority over all others.

#### Ward Floors.

If your ward floors have become porous through neglect and marked by tyreless wheel chairs, we fear that whatever polish you use and however much you polish them they will never look satisfactory. The damaged surface must first be removed. To get them planed by a carpenter would involve much expense in labour, and the result would be by no means perfect. It is practically impossible, even with the greatest skill, to get a perfectly even surface on a hardwood floor with an ordinary jack plane. We suggest that you get an estimate from the Simplex Floor Planing and Treatment Company, 23 City Road, Finsbury Pavement, London, E.C. 1. This firm makes a speciality of this kind of work, and by the use of electrically-driven planing and sand-papering machines they are able to get a perfectly level and smooth surface. The process is completed with other apparatus for filling cracks and polishing.—E. T. M.

### Question for January.

### UTILISING VOLUNTARY HELP

How would you deal with offers of voluntary part-time help in connection with your hospital from professional men, clerks, &c.? Give suggestions how their assistance might be utilised.

**A minimum payment of 10s. 6d. will be made for each published answer.)**

RULES.

The following rules must be observed:—

1. Contributions must be written on one side of the paper. Conciseness and terseness are desirable features. The MS. must bear the name and address of the sender and be accompanied by coupon to be cut from the back cover (inside page) of the current issue of The Hospital. A pseudonym must be chosen if the name is not to be published.

2. Contributions must be addressed to the Editor of The Hospital Institutional Worker Supplement, 28 & 29 Southampton Street, Strand, London, W.C. 2, should reach him before the end of the current month, and be marked in the left-hand corner "Facts and Figures."

### ENQUIRIES AND ANSWERS.

### THE SICK AND IN NEED.

### Assistance Required by Nurse for Emigration.

The King's Fund will not consider any applications for grants after January 31, but you can apply to either of the following for the help you require: The Overseas Settlement Committee, 59 Victoria Street, London, S.W. 1; and the Joint Committee at the same address.—Distressed.

### Farm Work—Instruction for Poor Boy.

Write to the Principal of the Gorsey Down Dairy Farm, Tatsfield, near Westerham, Kent, and inquire if there is a vacancy in the colony for the poor boy in question. The colony receives boys of all ages up to twenty, and instruction is given in all branches of farm work and gardening. Admission is by application to the Principal. Payment is expected according to circumstances.—B. F.

### Home of Rest for Clergyman.

You might write to the Rev. W. H. Oxley, 75 Victoria Road, W., who will inform you as to whether the St. John's House of Rest, Quartier de la Madone, Mentone, has been re-opened or not, and also if there is a vacancy. In the usual way the House is open from November 1 to May 1; the charge is £1 per week.—S. G. H.

### EMPLOYMENT AND TRAINING.

### Infirmary Training-Schools.

You might write to the Matrons of the following institutions: St. Leonard's Infirmary, 204 Hoxton Street, Shoreditch, London, N. (training four years, with C.M.B. certificate); St. Mary Abbott's (Kensington) Infirmary, Marloes Road, W. (four years' training, with C.M.B.); and St. Mary's (Islington) Infirmary, Highgate Hill, N.—Kitty.

### Address of Midwifery Associations.

The following are the addresses you ask for: Maternity Nursing Association, 63 Middleton Square, E.C. 1; Cottage Benefit Nursing Association, Denison House, Vauxhall Bridge Road, S.W. 1; Society for Promoting the Training of Midwives, Dacre House, Dean Farrar Street, Westminster, S.W. 1.—G. G.

### Training as Health Nurse.

We suggest that you write to the secretaries of the Battersea Polytechnic, London, S.W. 11; the National Health Society, 51 Berners Street, W. 1, and the Royal Sanitary Institute, Buckingham Palace Road, S.W. 1, for particulars of length of training and fees.—Trained Nurse.

